# AI-PLAX: AI-based placental assessment and examination using photos

**DOI:** 10.1016/j.compmedimag.2020.101744

**Published:** 2020-09

**Authors:** Yukun Chen, Zhuomin Zhang, Chenyan Wu, Dolzodmaa Davaasuren, Jeffery A. Goldstein, Alison D. Gernand, James Z. Wang

**Affiliations:** a The Pennsylvania State University, University Park, PN, USA; b Northwestern Memorial Hospital, Chicago, IL, USA

**Keywords:** Deep learning, Transfer learning, Placenta, Photo image analysis, Pathology

## Abstract

Post-delivery analysis of the placenta is useful for evaluating health risks of both the mother and baby. In the U.S., however, only about 20% of placentas are assessed by pathology exams, and placental data is often missed in pregnancy research because of the additional time, cost, and expertise needed. A computer-based tool that can be used in any delivery setting at the time of birth to provide an immediate and comprehensive placental assessment would have the potential to not only to improve health care, but also to radically improve medical knowledge. In this paper, we tackle the problem of automatic placental assessment and examination using photos. More concretely, we first address *morphological characterization*, which includes the tasks of placental image segmentation, umbilical cord insertion point localization, and maternal/fetal side classification. We also tackle *clinically meaningful feature analysis* of placentas, which comprises detection of retained placenta (*i.e.*, incomplete placenta), umbilical cord knot, meconium, abruption, chorioamnionitis, and hypercoiled cord, and categorization of umbilical cord insertion type. We curated a dataset consisting of approximately 1300 placenta images taken at Northwestern Memorial Hospital, with hand-labeled pixel-level segmentation map, cord insertion point and other information extracted from the associated pathology reports. We developed the AI-based Placental Assessment and Examination system (AI-PLAX), which is a novel two-stage photograph-based pipeline for fully automated analysis. In the first stage, we use three encoder-decoder convolutional neural networks with a shared encoder to address morphological characterization tasks by employing a transfer-learning training strategy. In the second stage, we employ distinct sub-models to solve different feature analysis tasks by using both the photograph and the output of the first stage. We evaluated the effectiveness of our pipeline by using the curated dataset as well as the pathology reports in the medical record. Through extensive experiments, we demonstrate our system is able to produce accurate morphological characterization and very promising performance on aforementioned feature analysis tasks, all of which may possess clinical impact and contribute to future pregnancy research. This work is the first for comprehensive, automated, computer-based placental analysis and will serve as a launchpad for potentially multiple future innovations.

## Highlights

• A novel two-stage pipeline for automated placental examination and assessment.
• A novel application of “similar/overlapped domains, different tasks” transfer learning.
• Curation of the first-of-its-kind large-scale dataset for placenta research.
• A fast and objective solution for placental measurement and assessment.
• Highly promising results for a number of diagnoses that may possess clinical impact.

## Graphical abstract

**Figure:**
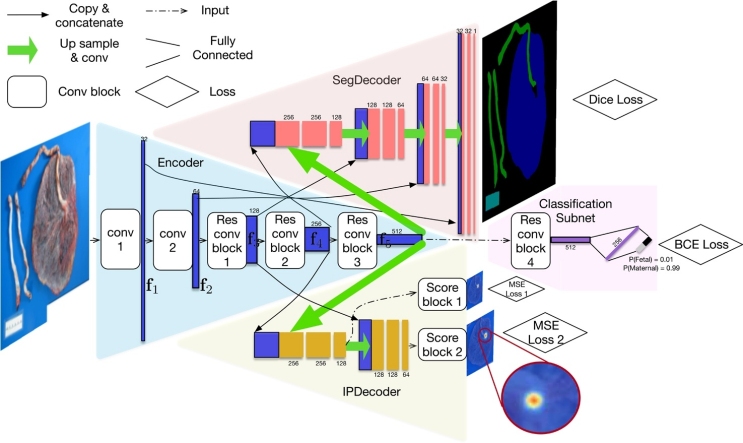

## 1 Introduction

**Fig. 1:**
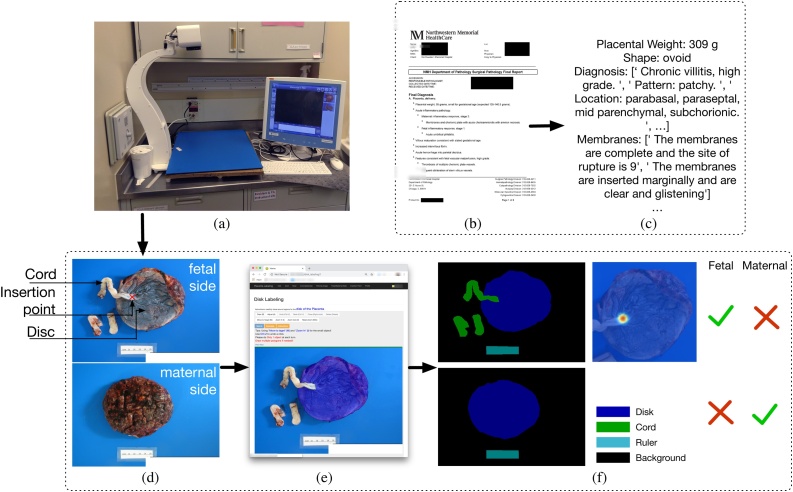
Data curation process. (a) Placenta photography equipment at Northwestern Memorial Hospital. This equipment is used to collect high and consistent quality placenta photos for the curation of our dataset. (b,c) Extracting diagnoses and measurements from de-identified pathological report in PDF format. (d–f) Collecting pixel-level segmentation map for cord, disc, and ruler, insertion point location, and whether an image captures fetal or maternal side placenta through our web-based labeling tool.

The placenta is a window into the events of a pregnancy and the health of the mother and baby (Roberts, 2008). Yet, a very small percentage of placentas around the world are ever examined by a pathologist. Even in developed countries like the U.S., placentas are examined and characterized by a pathologist only when it is considered necessary and resources are available. Full pathological examination is expensive and time consuming. Pathologists or pathologist assistants perform a macroscopic or gross examination and select sections for microscopic examination. After processing, they examine sections under a microscope and produce a written report (*e.g.*, Fig. 1(b) and (c)) that contains various measurements (*e.g.*, the weight, the disc diameter) and diagnoses (*e.g.*, completeness or retained placenta, cord insertion type, shape category, meconium, chorioamnionitis, etc.). In some specialty centers, including Northwestern, the gross examination includes photography using special equipment (Fig. 1(a)). These measurements and placental diagnoses can be useful for both short- and long-term clinical care of the mother and baby.

Automated placental assessment based on photographic imaging can potentially allow more placentas to be examined, reduce the number of normal placentas sent for full pathological examination, and provide more accurate and timely morphological and pathological measurements or analyses. Typical photographs of the placentas capture the umbilical cord inserting into the fetal side of the disc, as well as the maternal side appearance. Two example images of placentas can be found in Fig. 1(d). This paper focuses on a fully automated system for placental assessment and examination. Specifically, such systems will be responsible for placental segmentation, umbilical insertion point localization, fetal/maternal side classification, and the prediction of a number of pathological indicators (aka gross abnormality). These indicators include *retained placenta* (*i.e.*, incomplete placenta), *umbilical cord knot*, *meconium*, *abruption*, *chorioamnionitis*, *hypercoiled cord*, and *umbilical cord insertion type*. Some pathological findings from placentas are strictly microscopic; however, many have gross (macroscopic) and microscopic features, while some are only seen on gross exam. The latter are particularly frequent in placental pathology (Heerema-McKenney et al., 2019). Thus, we focus on predicting macroscopic pathological indicators.

### 1.1 Related work

Existing placental imaging research can be classified into two types based on the time the image is taken: *pre-delivery* and *post-delivery*. Because we cannot directly capture a photo for the placenta under visible light spectrum prior to the delivery, *pre-delivery* placental imaging research has been focused on images obtained through other means, *e.g.*, MRI (Alansary et al., 2016) and ultrasound (Malathi and Shanthi, 2011, Looney et al., 2017). Pre-delivery placental imaging research focuses on segmentation, which can be used as visual aids for doctors.

*Post-delivery* placental imaging research engages different methods and thus can be further categorized into two types: those using microscopic images of (Thomas et al., 2010, Kidron et al., 2017) and those using macroscopic images of the placenta taken by cameras (Yampolsky et al., 2009). A comprehensive overview of both microscopic and macroscopic placenta pathologies can be found in a book by Benirschke et al. (2012). While microscopic assessment is more established, it requires equipment and personnel to make slides and microscopes and microphotography to make images. In contrast, camera-based imaging in the second category only requires an ordinary camera or even a camera phone, and thus has greater potential to be widely adopted. We found a few prior works on macroscopic placental assessment from photos in the literature, but each focused on a specific aspect and involved human assessment as a part of the process. For example, Salafia et al. (2010), Yampolsky et al. (2008) studied variations in disc surface shape and vascular network from placental photos to identify associations between these factors and vascular pathologies and placental efficiency. Haeussner et al. (2013) attempted to estimate the size and shape of placentas from photos and found placenta size but not shape to have an association with the birth weight. To our knowledge, there has not been an automated approach to analyze placenta photographs. We believe such an approach has the potential for widespread adoption because today's smartphones have high-quality cameras as well as highly capable CPU, GPU, and/or AI chips.

**Fig. 2:**
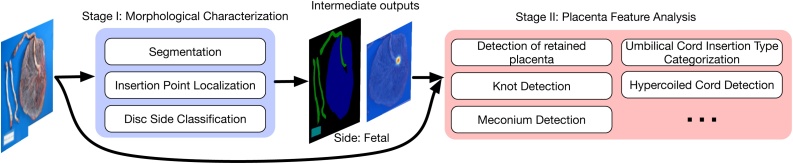
Schematic diagram of our proposed two-stage framework for automated placental assessment and examination using photos. Details of the Stage I and Stage II models will be described in Sections 2.2 and 2.3, respectively.

In this paper, we extend our earlier preliminary work (Chen et al., 2019) and present a two-stage pipeline (as illustrated in Fig. 2) for automated placental assessment and examination using photos. In the first stage (Stage I), we take a transfer learning (TL) approach to tackle the associated tasks of morphological characterization rather than employing an independent model for each task. Transfer learning promises performance gain and robustness enhancement through representation sharing for closely related tasks (Pan and Yang, 2009) and has become popular in medical imaging applications in recent years (Cheplygina et al., 2019). Cheplygina et al. (2019) summarizes the use of transfer learning into three categories: “same domain, different tasks”, “different domains, same task” and “different domains, different tasks”. Our method is closest to the “same domain, different tasks” category but is not an exact match. More precisely, our method should fall into a category described as “similar/overlapped domains, different tasks” because the source and target domains have overlap but are not the same (see Section 2.2 for more detailed discussions). Specifically, we transfer the learned representation of the encoder from the segmentation task to the other two tasks, *i.e.* disc side classification and insertion point localization. Our network architecture design takes inspiration from the recent deep learning advances on classification (He et al., 2016a), image segmentation (Long et al., 2015, Ronneberger et al., 2015), and key point localization (Newell et al., 2016). In particular, the design of our segmentation module follows the practice of concatenating feature maps in encoder with feature maps in decoder, such as performed in the U-Net (Ronneberger et al., 2015); and the design of our insertion point module follows the practice of regressing a Gaussian heat map, rather than using the coordinate values, as the ground truth, which has been shown to be successful in human key-point/joint localization tasks (Tompson et al., 2014, Benirschke et al., 2012, Newell et al., 2016, Payer et al., 2019). Tompson et al. (2014) first showed the importance of intermediate supervision to improving localization accuracy. We take their idea in our design by considering two heat map predictions in the final loss — one from the final feature layer and one from the intermediate feature layer. In the second stage (Stage II), we employ independent models each tailored for an individual task for a few important placental assessment tasks including but not limited to detection of *retained placenta* (*i.e.*, incomplete placenta), *umbilical cord knot*, *meconium*, *abruption*, *chorioamnionitis*, *hypercoiled cord*, and categorization of *umbilical cord insertion type*.

### 1.2 Rationale for a two-stage pipeline

We chose to pursue a two-stage pipeline based on the following observations, both of which make it difficult to build an end-to-end model for all tasks:

• Almost all of our second-stage tasks only apply to either the fetal side or the maternal side of a placenta or only to the disc/cord/ruler region.
• A relatively small fraction of all images bears the abnormalities we attempt to detect for the tasks in the second stage, and the sets of images bearing different abnormalities often have little overlap.

*The first observation* makes it natural for the second-stage tasks to take in the segmentation and disc-side predictions from the first stage to narrow down the region of interest and eliminate irrelevant information. Also, this means the input feature space for these tasks is rather different from the first stage or other second-stage tasks, and it is difficult, if not impossible, to apply transfer learning here to let those tasks benefit from the representations learnt from other tasks. In contrast, tasks in the first stage are more closely related and have larger overlapped input feature space. *The second observation* makes it sometimes impractical to use the same training/testing set for all tasks. Each task may have its own training/testing set such that the model will not be dominated by negative cases (*i.e.*, without abnormalities).

### 1.3 Contributions

We summarize the primary contributions as follows.

• We introduce a novel pipeline for comprehensive, automated placental assessment and examination using photos. The design of the pipeline, which has two stages, takes the relationship and the similarity of the tasks into consideration. Specifically, we use transfer learning to boost performance and robustness for closely related tasks with significant overlapped input space in the first stage. In the second stage, we use the first-stage predictions in separate models to address distinct tasks: to determine if an image is relevant (through side classification) and to provide the region of interest (through segmentation). Our method is explainable by design and achieves highly promising results. We believe isolating the models for irrelevant tasks and enforcing strong priors on the information flow between sub-models are critical under a limited label and robustness-prioritized setting, which is typical for medical image analysis. Such isolation is necessary to reduce the possibility of learning signals/correlations that do not hold true for the general distribution but just happen to be the case in our collected data based on prior domain knowledge. Additionally, distinct sub-models in the second stage can be developed in parallel and can be upgraded without worrying that it will affect performance for other tasks.
• Our use of transfer learning for the first-stage tasks can be categorized into the “similar/overlapped domains, different tasks” type, which is novel and can be applied to other medical image analysis problems.
• We curated a first-of-its-kind large-scale dataset with hand-labeled segmentation maps, umbilical cord insertion point location and diagnoses extracted from the associated pathology reports. This dataset enabled us to develop our computational pipeline addressing automated placental assessment and examination tasks. We believe the dataset will also be highly beneficial to future research on the placenta and adverse prenatal and postpartum outcomes.

## 2 Materials and methods

### 2.1 Dataset

We collected a dataset consisting of 18,400 placenta photos as well as the associated pathology reports written in natural English by the pathologist who originally examined the placenta, spanning the years of 2016–2018. The photos and reports are from Northwestern Memorial Hospital, a large urban academic medical center. The photos were taken by on-site pathologists and pathologist assistants using a camera installed on a fixed height arm against standardized blue background as illustrated in Fig. 1(a). Pathology classification is standardized, and the pathologists have perinatal training and expertise. From the 18,400 placenta photos (of about 9000 placentas), 1370 photos were selected to be hand labeled. 665 of the photos are fetal-side images, and 705 are maternal-side images.^2^ Fig. 1 shows our data curation process. We developed a web-based tool (Fig. 1(e)) to collect the following data: (i) the pixel-wise segmentation maps, (ii) the side-type label as fetal side or maternal side, and (iii) the cord insertion point (only for fetal side, visualized as a Gaussian heat map centered at the marked coordinate in (Fig. 1(f)) so that multiple trained labelers could annotate this dataset concurrently. We also extract diagnoses from the pathology reports (Fig. 1(b,c)). A complete list of diagnoses we extracted from the pathology reports are listed in Appendix A. For those placentas being diagnosed with being retained/incomplete the pixel-wise incomplete area was annotated by a highly-trained pathologist who is a research member (J.A.G.). For true knot in the cord, trained research members placed a bounding box around the knot with expert review as needed.

We divided the fully-labeled dataset into training and testing sets with the ratio of 0.8:0.2. Because the insertion point can only be observed from the fetal side, we only use the 665 fetal-side images for insertion point prediction, with the same training-testing ratio as aforementioned.

### 2.2 Stage I: Morphological characterization

**Fig. 3:**
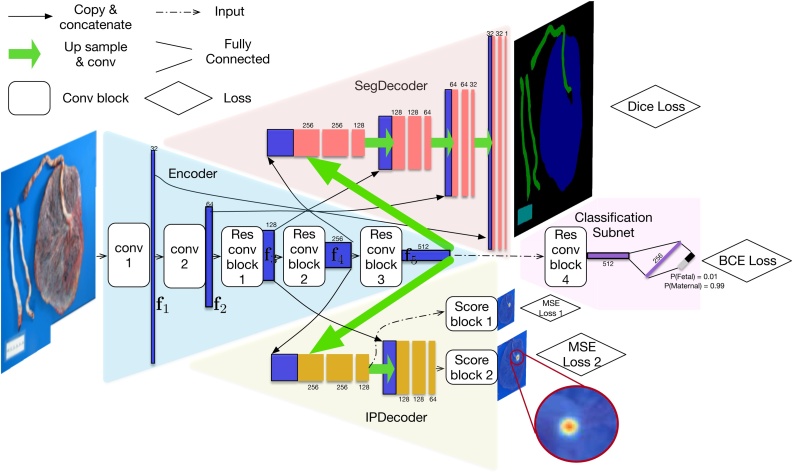
The architecture of our model for morphological characterization: a multi-head convolutional neural network for placenta image segmentation, cord insertion point localization, and placenta disc side classification. “Up sample & Conv” is implemented by a transposed convolution layer. “Res conv blocks” are residual blocks with two convolutional layers with stride 2 and 1, respectively, and the same kernel size 3 × 3. “Score blocks” are convolutional layers with kernel size 1 × 1 and the number of output channel 1. The soft-max layers are omitted. We use dice loss, binary cross entropy (BCE) loss and mean square error (MSE) loss for the segmentation, classification, and insertion point localization, respectively.

The proposed model for morphological characterization, as illustrated in Fig. 3, consists of an Encoder for feature pyramid extraction (blue), which is shared among all tasks, a fully convolutional SegDecoder for placenta image segmentation on both fetal- and maternal-side images (red), a Classification Subnet for fetal/maternal-side classification (purple), and a fully convolutional IPDecoder for insertion point localization.

#### 2.2.1 Encoder as feature pyramid extractor

The Encoder takes a placenta image **x** (either the fetal side or the maternal side) as the input and then outputs a pyramid of feature maps {**f**_1_, **f**_2_, **f**_3_, **f**_4_, **f**_5_} (represented as blue rectangles). Depending on the tasks, all or part of the feature maps are used by further task modules. Specifically, SegDecoder takes {**f**_1_, **f**_2_, **f**_3_, **f**_4_, **f**_5_} as input; Classification Subnet takes {**f**_5_} as input; and IPDecoder takes {**f**_3_, **f**_4_, **f**_5_} as input. The Conv-1 and Conv-2 blocks both consist of a Conv-BatchNorm-Relu layer. The difference, however, is that the Conv layer in the Conv-1 block has stride 1, while the Conv layer in Conv-2 block has stride 2. The Res conv blocks are residual blocks with two convolutional layers with stride 2 and 1, respectively, and the same kernel size 3 × 3, each of which spatially downsamples the input feature maps to half of its size and doubles the number of feature channels. The residual structure has been shown especially helpful for training deep architectures by He et al. (2016a).

#### 2.2.2 SegDecoder for segmentation

Our SegDecoder module consists of four expanding fully convolutional blocks, each of which takes the concatenation of a copy of the corresponding feature map **f**_*i*_, *i* ∈ {1, 2, 3, 4}, and transposes a convoluted (up-scaling factor 2) output feature map of the last layer. Finally, we apply soft-max to predict the probability of pixel (*i*, *j*) being of class *k*, denoted as **p**(*i*, *j*, *k*). To overcome the problem of highly imbalanced number of pixels for different categories, we use dice loss (Milletari et al., 2016) instead of the common cross entropy loss. Since we have four classes rather than two classes in Milletari et al. (2016), we adjust the dice loss to suit the 4-class scenario:

(1) $$L^{\mathrm{seg}}=1-\frac{\sum_{i,j}\sum_{k=0}^{3}\text{p}(i,j,k)\cdot \text{g}(i,j,k)}{\sum_{i,j}\sum_{k=0}^{3}({\text{p}}^{2}(i,j,k)+{\text{g}}^{2}(i,j,k))}\;,$$

where *i*, *j* run over the row and column indexes of an image, respectively; **p**(*i*, *j*, *k*) and **g**(*i*, *j*, *k*) denote the predicted probability of the pixel at location (*i*, *j*) and the 0/1 ground truth of that pixel belonging to class *k*, respectively.

#### 2.2.3 Classification Subnet for fetal/maternal side classification

Because the fetal/maternal side can be inferred from the “disc”region of a placenta alone, we crop the full placenta image **x** by a rectangle including the region of disc and resize the cropped image to 512 × 512 pixels as the input to the Encoder, which we denote as **x**_*c*_. The cropping is based on the ground truth segmentation map during training and on the predicted segmentation map at inference. Our Classification Subnet consists of a Res conv block, two fully connected layers, and a soft-max layer. At the end, a binary cross entropy (BCE) loss is applied to supervise the network.

#### 2.2.4 IPDecoder for insertion point localization

Because the insertion point is always located within or adjacent to the “disc” region, we use cropped disc region image **x**_*c*_, just as we perform cropping in Classification Subnet, as the input to the Encoder. Our IPDecoder is also fully convolutional and consists of two expanding fully convolutional blocks, the structure of which are the same as in the first two convolutional blocks in SegDecoder. The similarity of IPDecoder's structure with SegDecoder's helps us to ensure that the shared encoder representation could also be readily utilized here. Inspired by the success of intermediate supervision (Newell et al., 2016), we predict the insertion point localization heat map after each expanding convolutional block by a convolutional layer with kernel size 1 × 1 (denoted as “Score block” in Fig. 3) and use the MSE loss to measure the prediction error:

(2) $$L_{k}^{\mathrm{ip}}=\sum_{i,j}||\text{h}(i,j)-\hat{\text{h}}(i,j)|{|}^{2},\;\;k\in \{1,2\}\;,$$

where **h**(*i*, *j*) and $\hat{\text{h}}(i,j)$ are the ground truth (Gaussian) heat map and the predicted heat map, respectively. The final loss for insertion point is $L^{\mathrm{ip}}=L_{1}^{\mathrm{ip}}+L_{2}^{\mathrm{ip}}$. During inference, the predicted insertion point location is determined by $\left(i,j)={\arg\;\max}_{i,j}\hat{\text{h}}(i,j\right)$.

#### 2.2.5 Training and testing

We use mini-batched stochastic gradient descent (SGD) with learning rate 0.1, momentum 0.9, and weight decay 0.0005 for all training. We use a batch size of 2 for all segmentation training and a batch size of 10 for all insertion point localization and fetal/maternal side classification training. The procedures of training are as follows. We first train the SegDecoder + Encoder from scratch with parameters initialized to zero. Next, we fix the learned weights for the Encoder and train Classification Subnet and IPDecoder subsequently (in other words, the Encoder only acts as a fixed feature pyramid extractor at this stage). The rationale for making such choices is that the training for segmentation task consumes all images we have gathered and makes use of pixel-wise dense supervision, which is much less likely to lead to an overfitting problem. In contrast, the training for Classification Subnet takes binary value as ground truth for each image while the training for IPDecoder only uses around half of the whole dataset (only fetal-side images). To alleviate the lack of labels and to make the model more robust, we use common augmentation techniques including random rotation (±30°) as well as horizontal and vertical flipping for all training images.

#### 2.2.6 Implementation

We implemented the proposed pipeline in PyTorch (Steiner et al., 2019) and ran experiments on an NVIDIA TITAN Xp GPU. For segmentation training, all images are first resized to 768 × 1024, which is of the same aspect ratio as the original placenta images. For insertion point localization and fetal/maternal side classification training, we resize all cropped “disc” region images to 512 × 512, which is natural because the cropped “disc” regions often have a bounding box close to a square. We summarize all parameter settings for our model in Appendix B.

### 2.3 Stage II: placenta feature analysis

In this stage, we detect pathological indicators based on the results from Stage I.

#### 2.3.1 Detection of retained placenta

Retained placenta is a cause of postpartum hemorrhage, and if prolonged, it can serve as a nidus for infection (Silver, 2015). Trained birth attendants perform a focused examination of the placenta, including inspecting the maternal surface for completeness. However, this process may fail if there is not a trained birth attendant, if blood obscures incomplete areas, or if human error happens. Examination of placentas in pathology also includes assessment of the completeness of the maternal surface, which is recorded in the pathology report. The treatment for retained placenta includes removal of retained parts from the uterus. We identified 119 out of 705 maternal side placenta images in our dataset with possible “retained placenta” based on the pathology reports and we asked a perinatal pathologist (coauthor) to annotate where the possible missing parts are for each of the images. We trained two neural networks for this task, one for classification and one for localization.

The **classification** network is a binary classification CNN tasked with assessing if the placenta is retained (or incomplete) or not. As the incomplete parts are always within the disk region, the pixels out of the disk region are not considered for the binary classification and were excluded from the input. Thus, we use segmentation maps predicted in Stage I to extract the disk part of a placenta photo by setting pixels not classified as a part of the disc to zeros. Next, we feed the processed placenta photo into the classification network, which is a Resnet-18 network, chosen to suit the small scale of our training set. In training, we fine-tune on our dataset from a model pretrained on ImageNet (Deng et al., 2009) (with 1000 classes) using mini-batched stochastic gradient descent (SGD) with batch size 10, learning rate 0.01, momentum 0.9, and weight decay 0.0005 for all experiments.

The **localization** network assumes that the input placenta image has been classified as retained/incomplete and is tasked with segmenting out the retained/incomplete region(s). We treat it as a two-class segmentation problem and train our localization network, which we choose to be the Deeplab architecture (Chen et al., 2017) with ResNet-101 as the backbone network (pretrained on ImageNet (Deng et al., 2009)), against the expert-provided pixel-wise incomplete region labels. Segmentation map predicted in Stage I are used to exclude non-disc regions such that our localization network is not distracted by those pixels. The training set contains 57 images and the testing set contains 12 images. We use SGD with batch size 5, learning rate 0.01, momentum 0.9 and weight decay 0.0005.

#### 2.3.2 Umbilical cord insertion type categorization

**Fig. 4:**
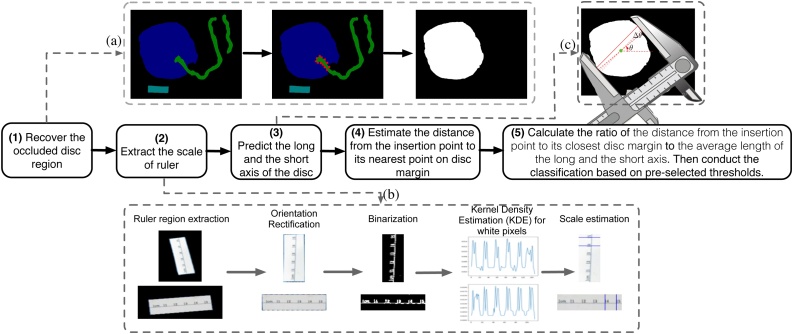
The insertion type categorization and related automated measurements procedures consists of steps (1)–(5). (a), (b), and (c) illustrate the detailed procedure for steps (1), (2), and (3), respectively.

Abnormal cord insertion is a feature of fetal vascular mal-perfusion (Khong et al., 2016). Based on the segmentation, the predicted insertion point location, and the scale we extracted from the ruler, we can measure the distance from the insertion point to the nearest margin of the disc, the length of the long- and short-axis of the disc (all in centimeters). Further, we classify the cord insertion type into “centrally”, “eccentrically”, and “marginally”, based on the ratio of *the distance from the insertion point to its closest disc margin* to *the average length of the long- and short-axis*. The thresholds for the above ratio between different categories are selected by optimizing classification accuracy on the training set. As illustrated in Fig. 4, the detailed procedures for insertion type categorization and related automated measurements are as follows.

1. We recover the occluded disc area by merging the originally predicted disc area with the polygon defined by vertices (red) adjacent with both disc area and cord area. Here, erosion and dilation image processing operations is used to remove small holes sometimes appearing in the disc region given by the raw segmentation prediction.
2. We extract the scale information from the ruler. Since the ruler in the image could be of any orientation, we first rectify the orientation of the ruler and fit a rectangle from the predicted ruler region. Next, we binarize the pixels within the ruler region such that the scale marker is more distinct. Thirdly, we use kernel density estimation to fit a distribution of the marker pixels (white after binarization) along the long edge of the ruler. Finally we read the number of pixels corresponding to one centimeter as the number of pixels between the two adjacent crests of the estimated distribution.
3. We estimate the long- and short-axis of a placenta by simulating how pathologists measure those from a 2-D shape by using a vernier caliper.
4. We estimate the distance from the insertion point to its nearest point on disc margin.
5. We calculate the ratio of *the distance from the insertion point to its closest disc margin* to *the average length of the long- and short-axis* and conduct the classification based on pre-selected thresholds based on optimizing training set classification accuracy.

#### 2.3.3 Meconium, abruption and chorioamnionitis detection

**Fig. 5:**
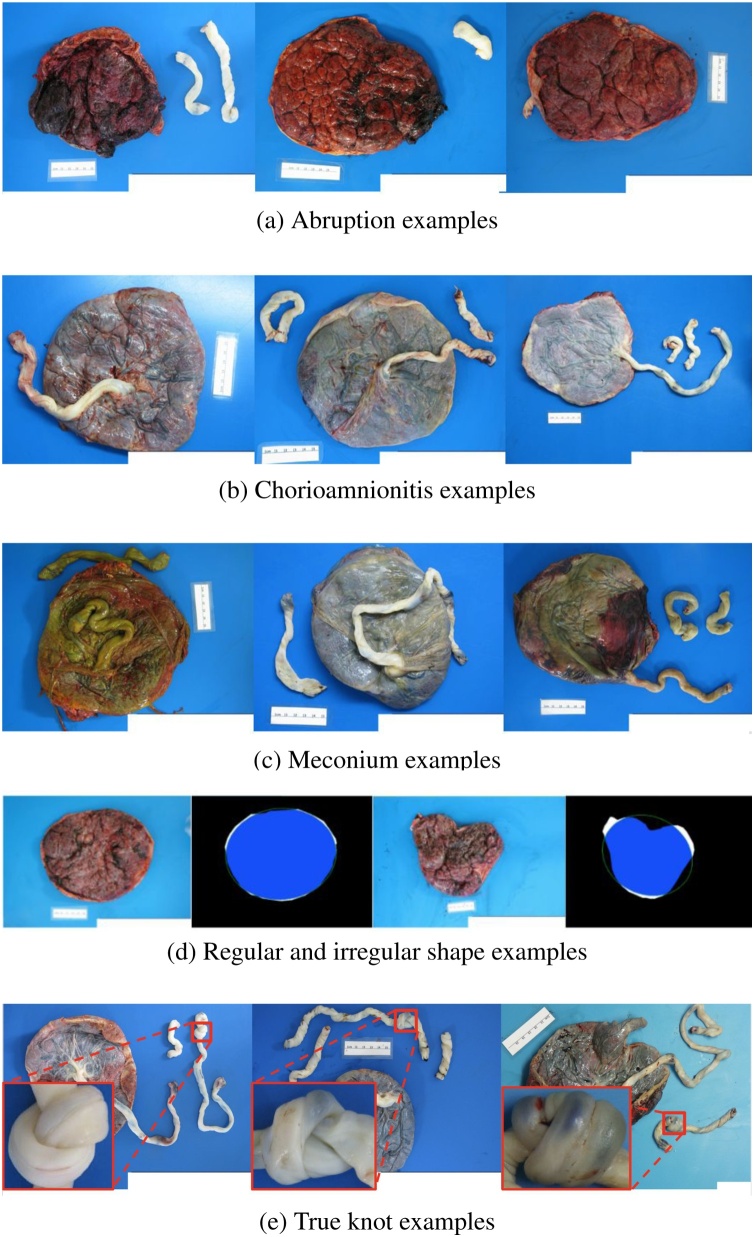
Sample placenta with abnormalities we aim to detect. Each row has a different abnormality. (a) Abruption (main indicator: blood clots on the maternal surface). (b) Chorioamnionitis (main indicator: opaque colored fetal surface). (c) Meconium (main indicator: meconium stain on the fetal surface). (d) Irregular shaped (left: regular, right: irregular. Their associated binary disc region along with the fitted ellipses are also displayed next to each of them). (e) True knots on the cord.

*Meconium* discharge is an indication of fetal distress and can damage the umbilical vessels as well as injure neonatal lungs (Kaspar et al., 2000). Meconium stains on the fetal membranes and/or the fetal surface of the placenta are seen in Fig. 5(a). Meconium is not always detectable from the gross color examination as shown in the third image (from left to right) of Fig. 5(a) and histological analysis is required in some cases. *Placental* abruption is separation of the placenta from the wall of the uterus before birth and can cause maternal blood loss and fetal distress or death. At delivery, dark red to brown adherent blood clots on the maternal side of placenta may be the main diagnostic characters of abruption; as seen in Fig. 5(b), however, this complication is not always visible. Larger clots suggest more severe abruption. *Chorioamnionitis* is an inflammation of the fetal membranes that often results from bacterial infection and which may progress to devastating infection of the fetus. The fetal surface of the placenta that is affected by chorioamnionitis often looks opaque, with the color ranging from white to yellow to green. The percentage of placenta images diagnosed with meconium, abruption, or chorioamnionitis are relatively low. As a consequence, the number in our fully labeled placenta images are too few for direct training of our model. To address this challenge, we build our training and testing set for these three tasks by using selected images of placentas diagnosed with these three problems out of the 18,400 images we collected in the year of 2016-2018. Specifically, we selected the set of images that satisfied our standards about freshness, non-placenta related objects in the image, etc. In sum, we used 470 meconium diagnosed fetal side images from a total of 731 cases, 268 chorioamnionitis diagnosed fetal side images from a total of 461 cases, and 181 maternal side images with abruption diagnosis from a total of 314 cases. For each task, we build the training and testing set by (1) randomly sampling the same amount of negative cases (not diagnosed with meconium, abruption or chorioamnionitis) as positive cases as found in the whole dataset; (2) splitting the whole assembled dataset into training and testing sets with the ratio of 0.8:0.2.

We trained one simple 6-layer convolutional neural network as the binary classifier for each of the three abnormalities. Only the disc region of an image is fed into those CNN classifiers and non-disc regions of the image are zeroing out based on our segmentation predictions. The first four layers are convolutional layers with filter size of 3, stride of 1, max pooling (for downsampling), relu activation and output sizes are 99 × 99 × 32, 48 × 48 × 64, 23 × 23 × 128, and 10 × 10 × 256, respectively. The last two layers are fully connected layers with 1024 neurons and 1 neuron, respectively. At the end, a sigmoid activation is used to scale the output in the range of [0, 1] as the probability for each class. We train each network for 30 epochs (until which the training loss has converged) using RMSProp optimizer with learning rate 0.001, momentum 0.9, batch size 10. Since abruption only appears on the maternal side and chorioamnionitis and meconium only appears on the fetal side, our classification network for each of them assumes a placenta image has already been classified into the associated side during inference.

#### 2.3.4 Irregular shape detection

Abnormal placental shape has been associated with premature birth or stillbirth (Salafia et al., 2010). The regular shape for a placenta is round or oval. Meanwhile, those placentas classified as irregularly shaped often looks star-like or calabash-like (as shown in Fig. 5(d)), with prominent concave or convex parts on the contour of the disc. By imitating how a pathologist determines if the shape of a placenta disc is irregular, we design a simple measure to quantify the irregularity of the disc shape for a placenta. First, we use the same module as in Section 2.3.2 (Fig. 4(1)) to recover the occluded disc and produce a whole disc region as a binary map. Next, we find the best-fit ellipse using zeroth-, first- and second-order moments. The (*p* + *q*)-th order moment is defined by:

(3) $$m_{p,q}=\iint x^{p}y^{q}f(x,y)\mathrm{dxdy},$$

where *f*(*x*, *y*) = 1 when the pixel is on the disc area, and zero otherwise. Then we can get the center coordinates (*x*_*c*_, *y*_*c*_), the inclination angle *α* and the long- and short-axis *a*, *b* of the ellipse following:

(4) $$x_{c}=\frac{m_{1,0}}{m_{0,0}},\;y_{c}=\frac{m_{0,1}}{m_{0,0}},$$

(5) $$\alpha =\frac{1}{2}\tan^{-1}\left(\frac{2m_{1,1}}{m_{2,0}-m_{0,2}}\right),$$

(6) $$a=\sqrt{\frac{2}{m_{0,0}}\left(m_{2,0}+m_{0,2}+{\left({\left(m_{2,0}-m_{0,2}\right)}^{2}+4m_{1,1}^{2}\right)}^{1/2}\right)},$$

(7) $$b=\sqrt{\frac{2}{m_{0,0}}\left(m_{2,0}+m_{0,2}-{\left({\left(m_{2,0}-m_{0,2}\right)}^{2}+4m_{1,1}^{2}\right)}^{1/2}\right)}.$$

Finally, we count the number of pixels covered by the fitted ellipse (denoted as *n*_1_), the number of disc pixels outside the fitted ellipse (denoted as *n*_2_), and the number of non-disc pixels within the ellipse (denoted as *n*_3_, those pixels are white ones in Fig. 5). We also define

(8) $$I=\frac{n_{2}+n_{3}}{n_{1}},$$

as the measure of irregularity for disc shape. Obviously, the larger the *I*, the more irregular a disc shape is. We select a threshold for *I* from the training set such that we classify a placenta as irregular-shaped if its *I* is larger than that threshold. Two examples of regular and irregular shaped placentas, along with their disc binary maps and fitted ellipses are displayed in Fig. 5(d).

#### 2.3.5 Hypercoiled cord identification

**Fig. 6:**
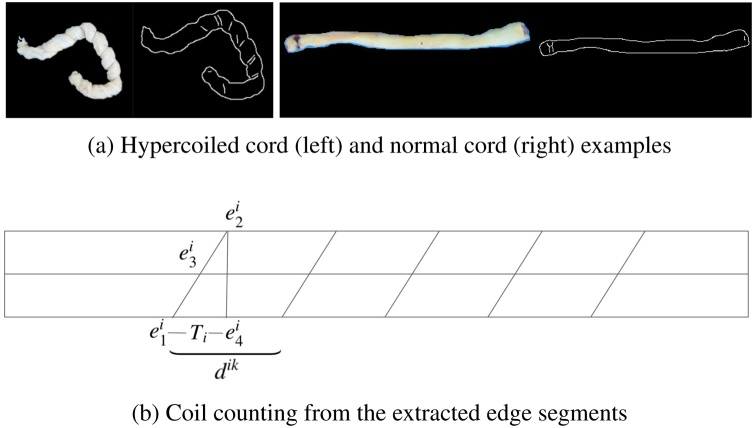
Fold crevice extraction and coil counting rules.

As illustrated in Fig. 6, a hypercoiled cord is more twisted than a normal cord, impairing fetal blood flow. Detecting this phenomenon is important because it is linked to infant mortality (Ernst et al., 2013). Our approach is to apply Canny edge detection on the cord region predicted by our segmentation model to detect fold crevices caused by hypercoiling. The count of those fold crevices could be a good approximation to the actual number of coils because it is also the main clue for pathologists to identify an individual coil and count the total number of coils using bare eyes. Before counting, we disregarded the detected fold crevices that are very small (in terms of length), crossed with the adjacent one, or whose orientation is too parallel with the orientation of the central skeleton of the cord. Fig. 6(a) shows two examples of our intermediate results for fold crevices detection. Sometimes, there are two or more edge segments extracted for one crevice, which will result in incorrect count of coils if we blindly count the number of extracted edge segments. We design a simple but effective rule, as illustrated in Fig. 6, to overcome this:

• Let $e_{1}^{i}$, $e_{2}^{i}$ and $e_{3}^{i}$be the points of intersection between the *i*th segment and the two cord boundaries and the central skeleton, respectively. Let $e_{4}^{i}$ be $e_{2}^{i}$'s projection (in the direction vertical to the central skeleton) onto the opposite boundary.
• Denote the length of the boundary between $e_{1}^{i}$ and $e_{4}^{i}$ as *T*_*i*_. Denote the distance between $e_{1}^{k}$ (*k* ≥ *i* + 1) of the *k*th segment and $e_{1}^{i}$ be *d*^*ik*^.
• If *d*^*ik*^ > 2*T*_*i*_, then the *k*th segment will be counted as a coil. Otherwise, the *k*th segment will not be counted.

Let us denote *n* the count of coils we obtain following the above rule and *l* the cord length in centimeters. We can quantify the coilness of a cord by:

(9) $$C=\frac{n\times 10}{l}\;,$$

*i.e.*, the number of coils per ten centimeters. After exploring the hypercoiled cords in the training set, we define a cord to be “hypercoiled” if *C* ≥ 4, which leads to the best training set accuracy when it is used as the classification criterion.

#### 2.3.6 Knot detection

A true knot forms when the umbilical cord ties around itself. Fig. 5(e) shows some examples of true knots. Loosely looped knots do not usually present a problem, while tight knots can severely affect the blood and oxygen exchange between the mother and the fetus. Therefore, knot detection is included as a routine examination by clinical staff at delivery and in the gross pathological exam. In a regular pathology report, a placenta is diagnosed with “normal” or “having true knots” or “having false knots” (which means the image does not contain true knot(s) but some part(s) of cords are very similar to true knots), In our dataset, we have 171 images diagnosed with having true knots and 462 images diagnosed with having false knots. For each placenta image diagnosed with having true knots, We manually labelled all the true knots with bounding boxes. Using these labeled images, we trained our knots detection module from scratch. For the knot detection task, we uses YOLO (Redmon et al., 2016), a single-stage detection network. we used the original RGB image concatenated with a binary mask denoting the cord region predicted by our segmentation as the input to the detection network and trained our detection network against the expert-labeled bounding boxes. As before, we used the 0.8:0.2 ratio to split the original dataset into training and testing sets. We used batch size 64 and learning rate 0.001.

## 3 Results

In this section, we summarize the experimental results using our dataset. The results are organized by the two stages and then by the individual tasks within each stage. We also discuss the inference time and the clinical significance at the end of this section.

### 3.1 Morphological characterization

#### 3.1.1 Segmentation

**Fig. 7:**
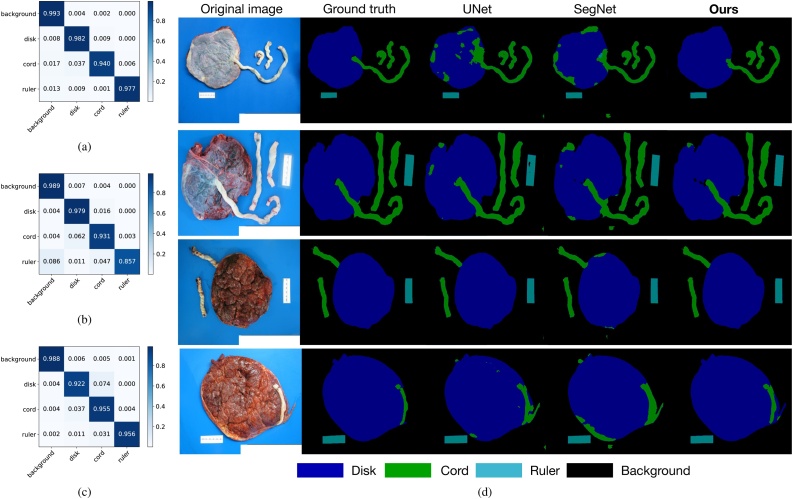
(a), (b), and (c) are confusion matrices of our approach, U-Net, and SegNet, respectively. (d) Examples of segmentation results. We show both fetal-side results (top two rows) and maternal-side results (bottom two rows).

We compared our approach with two fully convolutional encoder-decoder architectures, the U-Net (Ronneberger et al., 2015) and the SegNet (Badrinarayanan et al., 2017). The results are shown in Table 1 and Fig. 7.

**Table 1:** Segmentation evaluation.

Model	Pixel acc.	Class acc.	Mean IoU
U-Net	98.10	92.98	88.21
SegNet	96.51	94.56	84.57
ours	**98.73**	**97.26**	**93.93**

The best number in each column are highlighted with bold font.

We report the segmentation performance using standard segmentation metrics pixel accuracy, mean accuracy, and mean IoU. The definition of those metrics are as follows: suppose we have counted how many pixels are predicted to class *j* but with their ground truth being class *i* (for every *i*, *j* ∈ {0, 1, …, *k* − 1}, *k* is the number of classes) and we store it as the term **C**_*i*,*j*_ in a *k* × *k* matrix **C**. We also denote the (ground truth) total number of pixels for class *i* as *T*_*i*_. It is easy to see that $T_{i}=\sum_{j=0}^{k-1}{\text{C}}_{i,j}$. The pixel accuracy, mean class accuracy, and mean IoU are then defined as follows.

(10) Pixel accuracy:
  (10) $$\frac{\sum_{i=0}^{k-1}{\text{C}}_{i,i}}{\sum_{i=0}^{k-1}T_{i}}\;.$$
(11) Mean class accuracy:
  (11) $$\frac{1}{k}\frac{\sum_{i=0}^{k-1}{\text{C}}_{i,i}}{T_{i}}\;.$$
(12) Mean IoU:
  (12) $$\frac{1}{k}\sum_{i=0}^{k-1}\frac{{\text{C}}_{i,i}}{T_{i}+\sum_{j\neq i}{\text{C}}_{i,j}}\;.$$

In Fig. 7(a)–(c), we compare pixel-wise prediction confusion matrices of our approach, U-Net, and Segnet, respectively, which reflects more details about segmentation performance for different categories. We also show a few segmentation examples in Fig. 7(d) for qualitative comparison. Our approach yields the best segmentation results, especially for differentiating the cord and the ruler classes.

#### 3.1.2 Fetal/maternal side classification

**Fig. 8:**
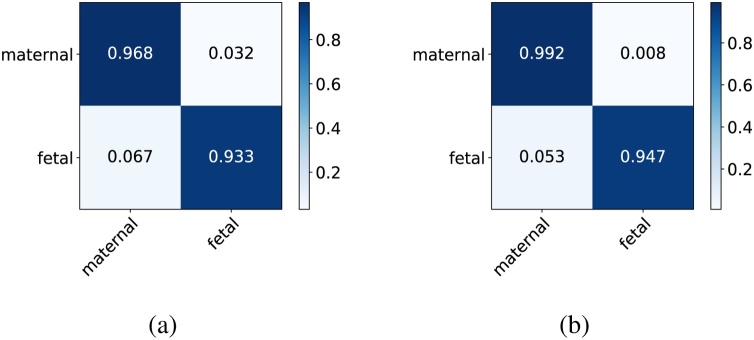
Fetal/maternal side classification confusion matrices comparison. (a) Without shared encoder weights. (b) Ours.

We achieved an overall fetal/maternal side classification accuracy of 97.51% on our test set. Without the shared encoder representation, we can only achieve 95.52% by training Encoder + Classification Subnet from scratch. We also compare their confusion matrices in Fig. 8.

#### 3.1.3 Insertion point localization

**Fig. 9:**
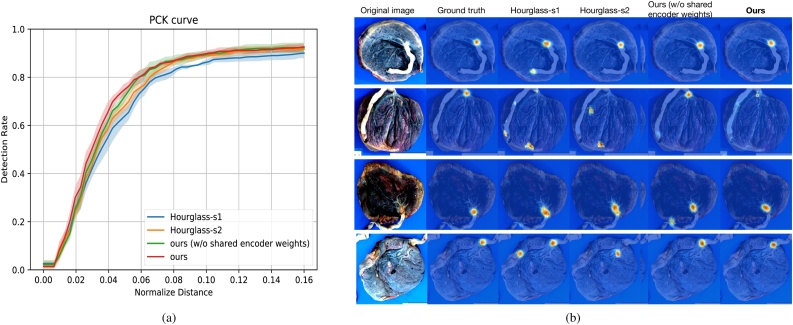
Evaluation of insertion point localization. (a) Quantitative evaluation with percentage of correct keypoints (PCK) curves. (b) Qualitative examples of insertion point heat map predictions.

We choose Percentage of Correct Keypoints (PCK) as the evaluation metric. PCK measures the percentage of the predictions fall within a circle of certain radius centered at the ground truth location. More formally, PCK at normalized distance *x* (*x* ∈ [0, 1]) is defined as:

(13) $$\mathrm{PCK}@x=\frac{\left|\{\text{p}:\frac{\sqrt{||\hat{\text{p}}-\text{p}|{|}_{2}}}{d}<x\wedge \text{p}\in {\left\{{\text{p}}_{i}\right\}}_{i=1}^{n}\}\right|}{n}\;,$$

where ${\left\{{\text{p}}_{i}\right\}}_{i=1}^{n}$ are the *n* keypoints we are trying to predict. $\hat{\text{p}}$ stands for our prediction for **p**; || . ||_2_ stands for the *L*-2 Euclidean distance and is used to measure the error of the prediction $\hat{\text{p}}$ from the ground truth **p**; | . | stands for the cardinality of a set. In our paper, we choose the diameter of the disc.^3^ as the normalizing factor *d*. In comparing our approach (both with and without shared encoder weights) to the Hourglass model (with number of stacks 1 and 2), we see competitive results achieved by our approach in human keypoint localization (Newell et al., 2016). Fig. 9(a) shows the PCK curves, with the *x* axis being the radius normalized by the diameter of the placenta. Each curve in Fig. 9(a) is the average of the results for five models trained with different seeds, and the light-colored band around each curve (view-able when the figure is enlarged) shows the standard deviation of the results. Our approach with shared Encoder consistently gives the best results, especially when the normalized distance is from 0.2 to 0.6. We also show a few qualitative examples of the insertion point heat maps predicted by each model, along with the ground truth in Fig. 9(b).

### 3.2 Placenta feature analysis

The predictions of the Stage I models enable us to conduct automatic placenta feature analysis by subsequent models/procedures.

#### 3.2.1 Detection of retained placenta

**Fig. 10:**
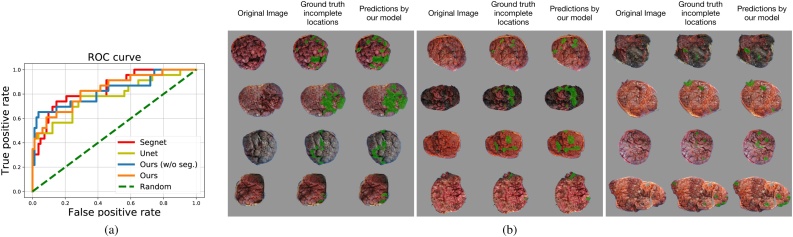
Quantitative and qualitative evaluation of the retained placenta classification and localization networks. (a) Receiver operating characteristic (ROC) curve for the classification network. (b) Qualitative examples of our incomplete part localization predictions produced by our localization network. (The figure is best to be viewed in color, as displayed in the web version of this article).

Both our classification network and localization network achieve promising results. We show the **r**eceiver **o**perating **c**haracteristic curve of the classification network in Fig. 10(a) and example localization results along with the ground truth in Fig. 10(b). To show the advantage of using the disc region only as the input, we compare two versions of classification network in Fig. 10(a), one with segmented disc region only (orange line, with AUC 0.836) and one without using our segmentation predictions (blue, with AUC 0.827). We also show the results of our classification network based on the disc regions provided by UNet (chartreuse, with AUC 0.781) and SegNet (red, with AUC 0.844) segmentation. The results based on our segmentation network in Stage-I is significantly better than the results based on UNet, and on par with or slightly worse than the results based on SegNet. We have expanded our pool of images with expert-labeled incomplete region (around 2×) from what we have for our conference paper (Chen et al., 2019) and improved our localization results from IOU = 0.571 to IOU = 0.636 by training on this expanded pool of labeled images. This improvement is also significant in our qualitative examples shown in Fig. 10(b).

#### 3.2.2 Umbilical cord insertion type categorization

**Fig. 11:**
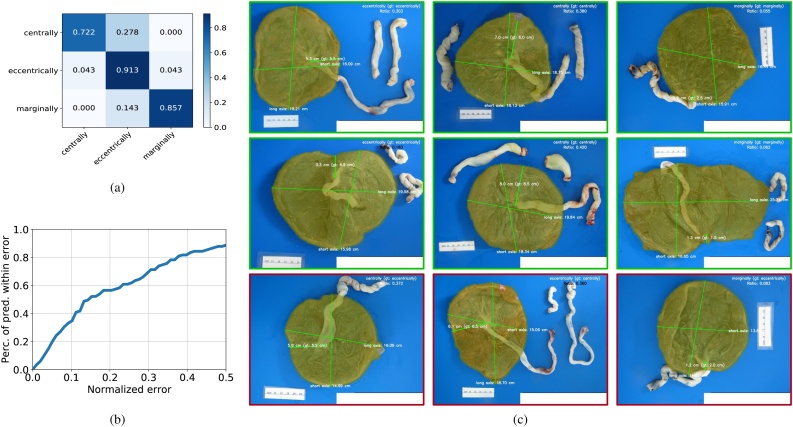
Quantitative and qualitative evaluation for insertion point type categorization. (a) The confusion matrix for insertion type categorization. (b) Quantitative evaluation of our estimation on the distance from the insertion point to the nearest disc margin. (c) Qualitative examples of insertion point type categorization. (The figure is best to be viewed in color, as displayed in the web version of this article).

We achieved an overall 88% test accuracy and we show the classification confusion matrix in Fig. 11(a). Because the ground truth *distance from the insertion point to its nearest point on the disc margin* can be extracted from the pathology reports, as shown in Appendix A, we are able to evaluate our prediction for this important intermediate value. Fig. 11(b) shows the evaluation for our estimation of *the distance from the insertion point to its nearest point on the disc margin* on the test set. The *x*-axis represents the threshold of the normalized error (absolute error normalized by the ground truth) and the *y*-axis shows the percentage of our estimation, the error of which is below such threshold. As shown, we have a 58% prediction accuracy if we set the threshold to 0.2. Qualitative examples of our insertion type categorization and associated automated categorization can be found in Fig. 11(c). Insertion type predictions are displayed in the upper right corner of each image, along with the ground truth in brackets. The success cases are green boxed and the failed cases are red boxed. For each image, the predicted insertion point location are marked with a green dot; a transparent green mask is overlaid on the image representing the predicted whole disc region; a (green) line is drawn between the insertion point and its nearest point on the disc margin. The predicted length of such line is displayed next to it, along with the ground truth length extracted from the pathology report (in brackets). The predicted long and short axes are also displayed, along with their predicted length in centimeters. We can see that the results for both the umbilical cord insertion type categorization and its related measurements are very appealing. Our method is already very promising as a replacement for the current approach based on the manual measurement and naked-eye inspection.

#### 3.2.3 Meconium, abruption, and chorioamnionitis detection

**Fig. 12:**
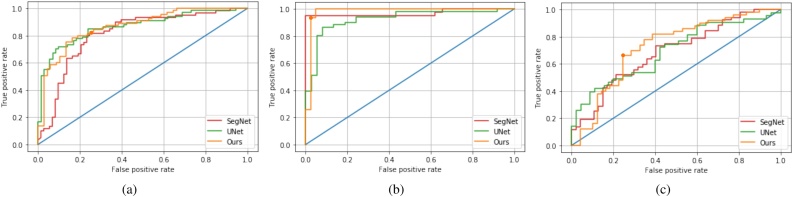
Receiver operating characteristic (ROC) curves for detecting meconium (a), abruption (b), and chorioamnionitis (c). (The figure is best to be viewed in color, as displayed in the web version of this article).

The ROC curves of binary classifiers for meconium, abruption, and chorioamnionitis are shown in Fig. 12(a)–(c), respectively. We achieved 0.97/0.98, 0.72/0.72 and 0.70/0.69 in terms of sensitivity and specificity for the detection of abruption, meconium, and chorioamnionitis, respectively, under the selected operating point marked on the ROC curve as shown in Fig. 12. We also show ROC curves of binary classifiers for meconium, abruption, and chorioamnionitis based on UNet and SegNet segmentations in each sub-figure. Overall, our segmentation network described in Stage-I is the best choice to achieve the best ROC curve for all three tasks.

#### 3.2.4 Irregular shape detection

In our dataset, 77 placentas are labeled as irregular shaped. By maximizing training set accuracy, we chose 0.14 as the irregularity measure (Eq. (8)) threshold for classifying the shape. The sensitivity and specificity for shape classification are 0.87 and 0.97, respectively, using the selected threshold. On expert review, the shape labels in pathology report are quite subjective, which we believe is the main limiting factor for achieving better classification performance in our model. We can, however, make the shape classification much more objective by switching from the current naked-eye inspection approach to our computer-based approach.

#### 3.2.5 Hypercoiled cord identification

Our dataset contains a total of 143 cords that are labeled as hypercoiled. The sensitivity and specificity for cord classification are 0.85 and 0.93, respectively, under the selected coilness threshold. We believe the results still have room for improvement. The main factors hindering our method from achieving better accuracy include blood stains within the image, faint edges on the cord, limited number of hypercoiled cases for selecting the threshold, and the cord segmentation prediction error.

#### 3.2.6 Knot detection

**Fig. 13:**
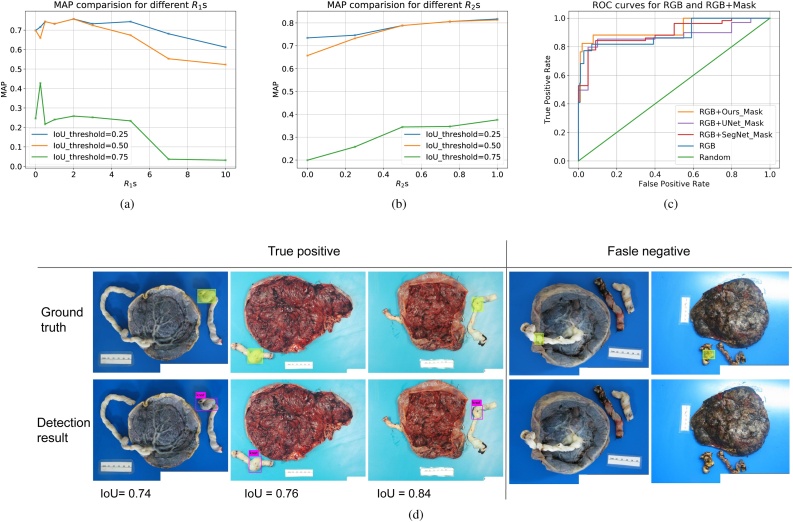
(a) The comparison of mean average accuracy (MAP) between different *ratios of the probability to sample an image with false knot or no knot over the probability to sample an image with true knot* (*R*_1_) under IoU threshold 0.25, 0.5 and 0.75. (b)The comparison of MAP between different *ratios of the probability to sample an image with false knot over the probability to sample an image with no knot* (*R*_2_) under IoU threshold 0.25, 0.5 and 0.75. Here, we assume *R*_1_ = 2, which is the best from results in (a). (c) The comparison of receiver operating characteristic (ROC) curves between using RGB only v.s. RGB+MASK as the input at IoU threshold 0.5. (d) Example detection results using RGB+MASK as input and *R*_1_ = 2 and *R*_2_ = 1.0. We also attach IoU value for each example here at the bottom. (The figure is best to be viewed in color, as displayed in the web version of this article).

We used the standard metric, mean average precision (MAP) under different thresholds of intersection over union (IoU) to evaluate our detection performance. In our dataset, the number of positive examples is significantly less than the number of negative examples and the number of hard negative examples (false knot) is significantly less than the number of easy negative examples (no knot). Such imbalance of different classes and imbalance of easy cases and hard cases could hurt the model's performance due to the dominating influence on the loss from the class in majority (or from the easy cases). This phenomenon has been verified and studied in many other applications and models, *e.g.*, (Lin et al., 2017, Cui et al., 2019). To address such a problem, we must balance the influence of different classes (or easy/hard cases) on the loss, either through an explicit re-weighting scheme by multiplying a scalar or implicit re-weighting scheme by adjusting the sampling for SGD. In that regard, we explored different sampling strategies instead of the default uniform sampling strategy when we use SGD to train our detection network. We present the results in Fig. 13(a) and (b). Specifically, we swept *the ratio of the probability of sampling an image with a false knot or no knot over the probability of sampling an image with a true knot* (*R*_1_) and *the ratio of the probability of sampling an image with a false knot over the probability of sampling an image with no knot* (*R*_2_). We then compare detection performance on the same test set under the training settings with different *R*_1_ (Fig. 13(a)) and different *R*_2_ (Fig. 13(b)). By default, if we sample uniformly from the training set, disregarding if a sample is positive/negative or is an easy/hard case, *R*_1_ = 7 and *R*_2_ = 0.5. We can see from Fig. 13(a) and (b) that we can achieve significantly better performance by decreasing *R*_2_ and increasing *R*_1_ from the default value, which translates to forcing our model attend more to negative cases (false knot or no knot), especially the hard negative cases (false knot). Under the best setting we selected (*R*_1_ = 2 and *R*_2_ = 1.0), we can achieve MAP 0.817, 0.813, 0.376 for IoU thresholds of 0.25, 0.5, and 0.75, respectively. Given the detection results by our model, we are able to classify whether an image has a true knot. And since classification itself is important in practice, we also show the ROC curve for our model from a binary classification perspective in Fig. 13(c) (orange line). As before, by concatenating the binary mask (given by our segmentation model in stage 1) for the cord with the original image's RGB channels, we achieve significant additional performance improvement. Quantitatively, we improved MAP from 0.77 to 0.81 and ROC curve from the blue line (AUC = 0.89) to the orange line (AUC = 0.93) in Fig. 13(c) by switching from RGB only to RGB+Ours_Mask as the input. Besides, when we concatenate the segmented masks provided by UNet and SegNet instead of the our segmentation network in Stage-I, the ROC curves (purple and red lines) become worse, and their AUC drop to 0.87 and 0.90, respectively. This again demonstrates the superior performance of our segmentation method. A few qualitative examples of true knot detection (our best model with *R*_1_ = 2 and *R*_2_ = 1.0 and using RGB+Ours_MASK as input) are shown in Fig. 13(d).

### 3.3 Inference time and discussion on the clinical significance

#### 3.3.1 Inference time

Table 2 summarizes the inference time of each individual component of our approach. For components not involving neural networks, we estimate the computation time by averaging over 10 images; for components involving neural networks accelerated by GPU, we estimate the computation time by averaging the inference time for 20 batches of images. Inference batch size used for each component is also displayed in Table 2. If we conduct segmentation for the maternal and fetal images at the same time and all other steps sequentially, the total inference time for a placenta is about 3.26 s. Moreover, if we parallelize the computation of *Side classification* and *Insertion point estimation* in Stage-I and all parallelizable components in Stage-II, the total inference time for a placenta is about 1.58 s. The inference time of the bottleneck components for the total inference time estimation are underlined in Table 2.

**Table 2:** Summary of inference time.

Component	Inference time (s./img.)	Batch size
Segmentation	$\underline{0.53}$	2
Side classification	0.09	10
Insertion point estimation	$\underline{0.18}$	10

Retained placenta classification	0.11	32
Retained placenta localization	0.47	10
Insertion type categorization	$\underline{0.87}$	NA
Meconium detection	0.19	16
Abruption detection	0.23	16
Chorioamnionitis detection	0.19	16
Irregular shape detection	0.39	NA
Hypercoiled cord identification	0.31	NA
Knot detection	0.28	10

The underlined time are the bottleneck bottleneck components' for overall inference time.

#### 3.3.2 Discussion on clinical significance

Our approach can significantly reduce the work burden of clinicians. Currently it takes about 15 min for a trained physician at Northwestern Memorial Hospital to examine the placenta and produce a pathology report that covers all diagnoses tackled by our approach, according to the perinatal pathologist (coauthor) in our team. This is about 276 (569) times of the inference time of the sequential (parallel) version of our approach.

More importantly, the benefits of a fully automatic system is not limited to faster inference time. Other benefits of our approach include:

• High objectivity: There can be inconsistent diagnoses among a group of physicians or even with the same physician over time. Our approach, however, always predicts using the same set of criteria and is a deterministic process.
• 24/7 availability and flexibility: For instance, if a woman delivers on Saturday at noon, the placenta would not even make it to the pathology lab until the next Monday morning. In contrast, our approach can provides timely on-site diagnoses so prompt treatment can be given to the mother and/or the baby if necessary.
• Scalability: By deploying our system in cloud services, we can use more machines when the demand is high. In contrast, it is costly to train and employ pathologists to meet sudden higher demand of the service.

We do want to emphasize, however, that the system has much room to improve further before it can be fully deployed clinically. Example areas to improve are as follows:

• We need to develop an easy-to-use interface for the physicians so they can easily run our algorithms, view, search, compare, and correct results generated by our system, integrate it into their current or updated workflow, and provide specific feedback. We have already developed a prototype demo, specifically, a Web application running all our algorithms at the back-end. Please see the supplementary video file. But there is still a gap between our prototype and a clinical software/system.
• We need to collect feedback from physicians and pathologists. There may be some cases that our system fails to predict due to limited training data or design flaw that we may not be aware of.

These problems are already beyond the scope of this paper and we will tackle them in the future works. Before overcoming these obstacles, we believe deploying our approach for triage purpose in clinical setting is the most promising first step. This will help hospitals prioritize limited pathology resources toward most needed cases, and at the same time minimize the health risk of patients using our automatic approach. Clinical adoption of our technology will be a gradual process that include a few phases/iterations but not a once-for-all feat.

## 4 Conclusion & discussion

We proposed a two-stage pipeline to address the tasks for automated placental assessment and examination. *In the first stage*, we designed a compact multi-head encoder-decoder CNN to jointly solve morphological placental characterization tasks by employing a transfer learning training strategy. We showed that our approach can achieve better performance than competitive baselines for each task. We also showed that the representation learned from the segmentation task can benefit insertion point localization and fetal/maternal side classification task. *In the second stage*, we used the output from the first stage, as well as the original placenta photos, as the input and employed multiple independent models for a few noteworthy placental assessment tasks. Through ablation experiments, we demonstrated that the predictions from the first stage models help us achieve better performance for tasks in this stage. For second-stage placenta feature analysis tasks, though our results still have room to be improved, especially when more placental images diagnosed with those abnormalities are available in the future, our current approaches are already useful for triage purpose, which could significantly alleviate the workload for pathologists.

In the future, it will be interesting to explore if some of these tasks can benefit from both fully-labeled (small fraction) and unlabeled placenta (large fraction) photos by using semi-supervised learning techniques. Automated prediction of additional and potentially more fine-grained pathological indicators beyond the ones tackled in this paper is also a direction we will pursue. We believe the prediction of some of those indicators could benefit from the predictions from the two stages of our current pipeline, such that a multi-stage pipeline becomes feasible.

## Authors’ contribution

Yukun Chen: methodology, software, data curation, writing – original draft, visualization. Zhuomin Zhang: methodology, software, data curation, writing – original draft, visualization. Chenyan Wu: methodology, software, data curation, writing – original draft, visualization. Dolzodmaa Davaasuren: methodology, software, data curation, writing – original draft, visualization. Jeffery A. Goldstein: conceptualization, data curation, resources, writing - review & editing, validation. Alison D. Gernand: conceptualization, resources, writing – review & editing, supervision, project administration, funding acquisition. James Z. Wang: conceptualization, resources, writing – review & editing, supervision, project administration, funding acquisition.

## Conflict of interest

None declared.

## Figures and Tables

**Table A1:** Information extracted from pathology reports.

Category	Item	Description in the pathology report
General informatiron	Image ID	Unique ID given to the image
	Maternal age	Age of the mother at the time of delivery
	Gestational age	Age of fetus at the time of delivery
	Placental weight	Weight of placenta in grams
	Delivery type	Cesarean or vaginal
	Freshness	Fresh or Fixed
	Shape	Age of the mother at the time of delivery
Cord	Knot	True, false or none
	Coils	Number of twists/coils in the segment of cord
	Diameter	Average diameter, Minimum and Maximum diameter
	Insertion type	Central, eccentric, margina, velamentous, furcate
	Insertion distance	Distance from the cord insertion to the nearest margin
Membrane	Color	Opaque, Green, yellow, clear, pink, pruple, tan, gray
	Insertion type	Circummarginate, circumvallate, marginal
Fetal side	Color	Opaque, green, red, blue, yellow, clear, Pink, purple, tan, gray, white, variegated
Maternal side	Completeness	Complete, incomplete, fragmented, Disrupted, ragged, roughened
	Adherent blood clots	Adherent blood clot observed in the gross description
Final diagnosis	Meconium	Meconium is detected in the final diagnosis
	Amnion nodosum	Amnion nodosum is detected in the final diagnosis
	Accessory lobe	Accessory lobe is detected in the final diagnosis
	Chorangioma	Chorangioma is detected in the final diagnosis
	Abruption	Abruption is detected in the final diagnosis
	Chorioamnionitis	Chorioamnionitis is detected in the final diagnosis
	Placental hypoplasia	Placental hypoplasia is detected in the final diagnosis

**Table B1:** Summary of parameter settings.

Parameter	value
Input image dimensions	768 × 1024 (Encoder+SegDecoder)
	512 × 512 (Encoder+Classification SubNet)
	512 × 512 (Encoder+IPDecoder)
Learning rate	0.1
Momentum	0.9
Weight decay	0.0005
Batch size	2 (Encoder+SegDecoder)
	10 (Encoder+Classification SubNet)
	10 (Encoder+IPDecoder)
Epochs	10

## Appendix A Information extracted from pathology reports

Table A1 summarizes the information we extracted from a pathology report.

## Appendix B Summary of parameter settings

Table B1 summarizes the all parameter settings for our Stage-I model.

## Footnotes

2 The numbers of fetal-side and maternal-side images are uneven because some of the collected images did not meet our image quality standard (*e.g.*, disc occluded by irrelevant object such as scissors) and we had to discard them from the dataset. We plan to release our dataset in the future after substantial expansion.

3 In practice, we approximate the diameter of the disc by the distance between the right most and left most pixel of the “disc” area in the segmentation map.

## References

[bib0005] Alansary A., Kamnitsas K., Davidson A., Khlebnikov R., Rajchl M., Malamateniou C., Rutherford M., Hajnal J.V., Glocker B., Rueckert D. (2016). Fast fully automatic segmentation of the human placenta from motion corrupted MRI. Proceedings of the International Conference on Medical Image Computing and Computer-Assisted Intervention.

[bib0010] Badrinarayanan V., Kendall A., Cipolla R. (2017). Segnet: A deep convolutional encoder-decoder architecture for image segmentation. IEEE Trans. Pattern Anal. Mach. Intell..

[bib0015] Benirschke K., Burton G.J., Baergen R.N. (2012). Pathology of the Human Placenta.

[bib0020] Chen L.-C., Papandreou G., Kokkinos I., Murphy K., Yuille A.L. (2017). Deeplab: Semantic image segmentation with deep convolutional nets, atrous convolution, and fully connected CRFS. IEEE Trans. Pattern Anal. Mach. Intell..

[bib0025] Chen Y., Wu C., Zhang Z., Goldstein J.A., Gernand A.D., Wang J.Z. (2019). Placentanet: Automatic morphological characterization of placenta photos with deep learning. Proceedings of the International Conference on Medical Image Computing and Computer-Assisted Intervention.

[bib0030] Cheplygina V., de Bruijne M., Pluim J.P. (2019). Not-so-supervised: A survey of semi-supervised, multi-instance, and transfer learning in medical image analysis. Med. Image Anal..

[bib0035] Cui Y., Jia M., Lin T.-Y., Song Y., Belongie S. (2019). Class-balanced loss based on effective number of samples. Proceedings of the IEEE Conference on Computer Vision and Pattern Recognition.

[bib0040] Deng J., Dong W., Socher R., Li L.-J., Li K., Fei-Fei L. (2009). Imagenet: A large-scale hierarchical image database. Proceedings of the IEEE Conference on Computer Vision and Pattern Recognition.

[bib0045] Ernst L.M., Minturn L., Huang M., Curry E., Su E. (2013). Gross patterns of umbilical cord coiling: correlations with placental histology and stillbirth. Placenta.

[bib0050] Haeussner E., Schmitz C., Von Koch F., Frank H.-G. (2013). Birth weight correlates with size but not shape of the normal human placenta. Placenta.

[bib0055] He K., Zhang X., Ren S., Sun J. (2016). Deep residual learning for image recognition. Proceedings of the IEEE Conference on Computer Vision and Pattern Recognition.

[bib0060] Heerema-McKenney A., Popek E.J., De Paepe M. (2019). Diagnostic Pathology: Placenta E-Book.

[bib0065] Kaspar H., Abu-Musa A., Hannoun A., Seoud M., Shammas M., Usta I., Khalil A. (2000). The placenta in meconium staining: lesions and early neonatal outcome. Clin. Exp. Obstet. Gynecol..

[bib0070] Khong T.Y., Mooney E.E., Ariel I., Balmus N.C., Boyd T.K., Brundler M.-A., Derricott H., Evans M.J., Faye-Petersen O.M., Gillan J.E. (2016). Sampling and definitions of placental lesions: Amsterdam placental workshop group consensus statement. Arch. Pathol. Lab. Med..

[bib0075] Kidron D., Vainer I., Fisher Y., Sharony R. (2017). Automated image analysis of placental villi and syncytial knots in histological sections. Placenta.

[bib0080] Lin T.-Y., Goyal P., Girshick R., He K., Dollár P. (2017). Focal loss for dense object detection.. Proceedings of the IEEE International Conference on Computer Vision.

[bib0085] Long J., Shelhamer E., Darrell T. (2015). Fully convolutional networks for semantic segmentation. Proceedings of the IEEE Conference on Computer Vision and Pattern Recognition.

[bib0090] Looney P., Stevenson G.N., Nicolaides K.H., Plasencia W., Molloholli M., Natsis S., Collins S.L. (2017). Automatic 3D ultrasound segmentation of the first trimester placenta using deep learning. IEEE International Symposium on Biomedical Imaging.

[bib0095] Malathi G., Shanthi V. (2011). Statistical measurement of ultrasound placenta images complicated by gestational diabetes mellitus using segmentation approach. J. Inform. Hiding Multimedia Signal Process..

[bib0100] Milletari F., Navab N., Ahmadi S.-A. (2016). V-Net: fully convolutional neural networks for volumetric medical image segmentation. Proceedings of the International Conference on 3D Vision (3DV).

[bib0105] Newell A., Yang K., Deng J. (2016). Stacked hourglass networks for human pose estimation. Proceedings of the European Conference on Computer Vision.

[bib0110] Pan S.J., Yang Q. (2009). A survey on transfer learning. IEEE Trans. Knowl. Data Eng..

[bib0115] Payer C., Štern D., Bischof H., Urschler M. (2019). Integrating spatial configuration into heatmap regression based CNNs for landmark localization. Med. Image Anal..

[bib0120] Redmon J., Divvala S., Girshick R., Farhadi A. (2016). You only look once: unified, real-time object detection. Proceedings of the IEEE Conference on Computer Vision and Pattern Recognition.

[bib0125] Roberts D.J. (2008). Placental pathology, a survival guide. Arch. Pathol. Lab. Med..

[bib0130] Ronneberger O., Fischer P., Brox T. (2015). U-Net: convolutional networks for biomedical image segmentation. Proceedings of the International Conference on Medical Image Computing and Computer-Assisted Intervention.

[bib0135] Salafia C.M., Yampolsky M., Misra D.P., Shlakhter O., Haas D., Eucker B., Thorp J. (2010). Placental surface shape, function, and effects of maternal and fetal vascular pathology. Placenta.

[bib0140] Silver R.M. (2015). Abnormal placentation: placenta previa, vasa previa, and placenta accreta. Obstet. Gynecol..

[bib0145] Steiner B., DeVito Z., Chintala S., Gross S., Paszke A., Massa F., Lerer A., Chanan G., Lin Z., Yang E., Desmaison A., Tejani A., Kopf A., Bradbury J., Antiga L., Raison M., Gimelshein N., Chilamkurthy S., Killeen T., Fang L., Bai J. (2019). Pytorch: an imperative style, high-performance deep learning library.. Advances in Neural Information Processing Systems.

[bib0150] Thomas K.A., Sottile M.J., Salafia C.M. (2010). Unsupervised segmentation for inflammation detection in histopathology images. Proceedings of the International Conference on Image and Signal Processing.

[bib0155] Tompson J.J., Jain A., LeCun Y., Bregler C. (2014). Joint training of a convolutional network and a graphical model for human pose estimation. Advances in Neural Information Processing Systems.

[bib0160] Yampolsky M., Salafia C.M., Shlakhter O., Haas D., Eucker B., Thorp J. (2008). Modeling the variability of shapes of a human placenta. Placenta.

[bib0165] Yampolsky M., Salafia C.M., Shlakhter O., Haas D., Eucker B., Thorp J. (2009). Centrality of the umbilical cord insertion in a human placenta influences the placental efficiency. Placenta.

